# Mechanically Robust Photo-responsive Liquid Crystal Elastomers

**DOI:** 10.1021/acsomega.6c02486

**Published:** 2026-04-09

**Authors:** Xi-tong Dong, Kuo-huai Qin, Hao Ouyang, Zhi-Hui Ren, Zheng-Hui Guan

**Affiliations:** Key Laboratory of Synthetic and Natural Functional Molecule of the Ministry of Education, Department of Chemistry & Materials Science, 12657 Northwest University, Xi’an 710127, P. R. China

## Abstract

Photoresponsive liquid crystal elastomers (LCEs) are promising for applications in soft robotics and smart devices owing to their precise controllability and excellent stability. However, their practical utility is often hampered by limitations such as notably inferior mechanical properties, a single shape memory effect, and inadequate recyclability. To address these challenges, we developed a photoresponsive liquid crystal-polyurethane interpenetrating polymer network (IPN-0.75) that exhibits outstanding mechanical performance, including an ultrahigh tensile stress of 44 MPa and a fracture strain of 1699%. The material also demonstrated excellent recyclability, retaining 93% of its original tensile strength after five recycling cycles. Moreover, IPN-0.75 shows remarkable photostability with a consistent response time (∼8 s) and a maximum bending angle (∼85°) over 10 actuation cycles. By integration of a triple shape memory effect and overcoming the mechanical weaknesses of conventional LCEs, this work opens up new possibilities for advanced applications in adaptive and sustainable soft actuators.

## Graphical abstract

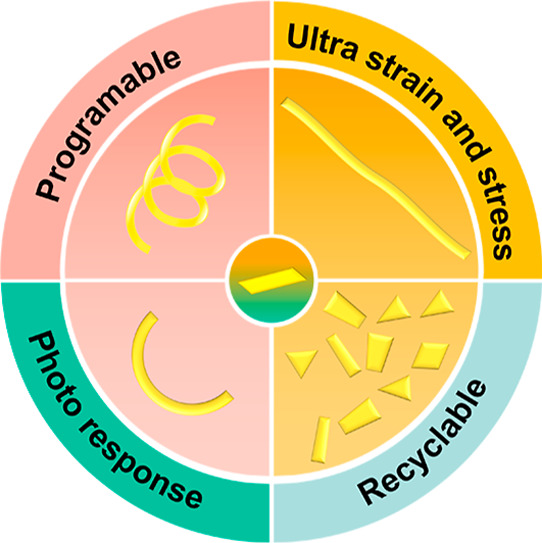

## 1 Introduction

Photoresponsive liquid crystal elastomers (LCEs), as a prominent class of photoresponsive actuator (PRA) materials,^1^ are distinguished by their high controllability and excellent stability. These properties make them highly suitable for advanced applications in fields such as flexible robotics,^2,3^ biomedicine,^4,5^ and smart devices.^6,7^ However, their practical adoption in broader industrial applications remains limited.^8–10^ This limitation is primarily due to inherent material drawbacks, including inadequate recyclability and insufficient mechanical properties, specifically low tensile stress and strain. Consequently, enhancing the mechanical performance of LCEs represents a critical and ongoing research challenge.

To address the limitations in mechanical properties and recyclability of LCEs, several enhancement strategies have been developed, primarily based on supramolecular cross-linking and chain entanglement. For instance, the incorporation of ureidopyrimidinone (UPy) groups enables the formation of quadruple hydrogen bonds, leading to a tensile stress of 8.5 MPa and a strain of 50%.^11,12^ Alternatively, mechanical kneading has been employed to promote chain entanglement, increasing the cross-linking density and achieving a tensile stress of 2.5 MPa with a strain of 400%.^13^ However, these approaches, which focus solely on modifying the single LCE network, still fall short of attaining an optimal balance between the tensile stress and strain. As a result, research interest has shifted to dual-network polymer systems.^14^ As a developing dual-network system, the interpenetrating polymer network (IPN) has been regarded as an effective way to enhance the mechanical properties of polymer materials. Constituted by the interweaving of two networks, each component of the IPN interpenetrates and entangles the others. The properties of each component complement one another through entanglement, thereby avoiding the defects inherent in single-network systems. Building on this concept, interpenetrating a mechanically robust polymer network with a relatively weaker component is an effective strategy to enhance the mechanical performance. This approach has been demonstrated to be effective in previous studies.^15,17^

In this work, an interpenetrating polymer network composed of polyurethane (PU) and poly­(acrylate) (PA) was successfully prepared.^16,18,19^ The PU network is designed to provide exceptional mechanical robustness and recyclability, achieving a remarkable tensile stress of 44 MPa and a fracture strain of 1699%. Simultaneously, the PA network incorporates liquid crystalline order and azobenzene units, endowing the material with photoresponsive actuation. Additionally, the unique phase transition exhibited by LCE endows the IPN elastomers with three-shaped memory effects.^20–24^ This advanced soft material displays considerable potential for applications in the domains of flexible robotics and bionic devices.

## 2 Results and Discussion

### 2.1 Preparation and Characterization of IPN-X Elastomers

The IPN comprises two components: LCE and PU. The LCE network was formulated using the mesogenic LC monomer 1,4-bis-[4-(3-acryloyloxypropyloxy)­benzoyloxy]-2-methylbenzene (RM257), the photoresponsive unit 4,4′-bis­[6-(acryloyloxy)­hexyloxy]­azobenzene (D6AB), chain extender 3,6-dioxa-1,8-octanedithiol (EDDET), and photoinitiator phenylbis­(2,4,6-trimethylbenzoyl)­phosphine oxide (BAPO). The composition of PU includes 4,4′-methylenedicyclohexyl diisocyanate (HMDI), soft segment poly­(tetrahydrofuran) (PTMEG ∼2000), hard segment isophthalic dihydrazide (IPDH), 4,4′-ethylenedianiline (EDA), 4,4′-dihydroxydiphenyl disulfide (OPDS), and the catalyst dibutyltin dilaurate (DBTDL), as schematically illustrated in Figure 1b. For comparison, pure LCE and PU films were also prepared under equivalent conditions. Detailed preparation steps and formulations are provided in the Supporting Information.

**Figure 1:**
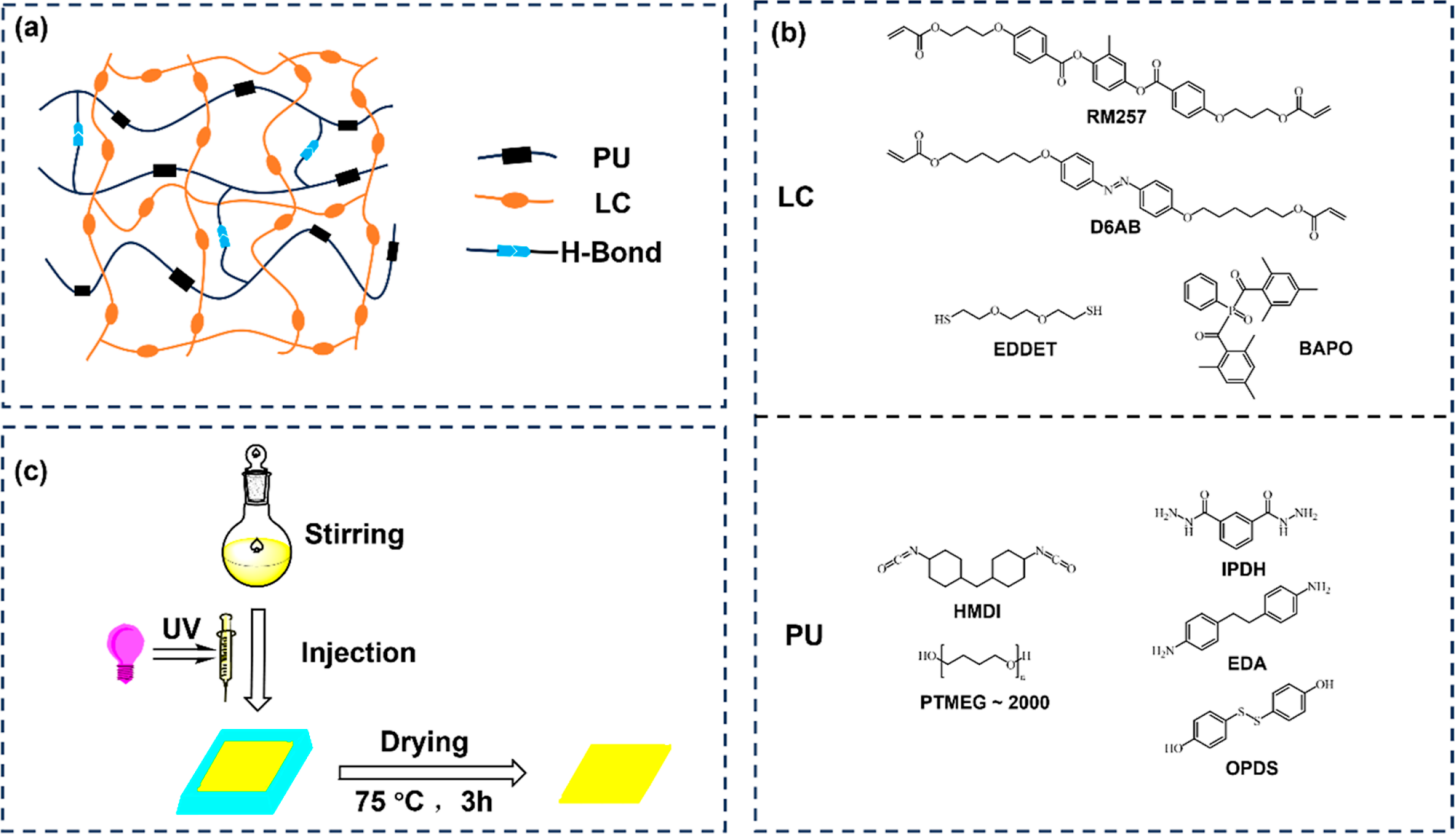
(a) Structure of IPN-X elastomers. (b) Composition of LCE and PU. (c) Preparation steps for IPN-X elastomers.

The successful preparation of the photoactive unit D6AB and IPN-X elastomers was confirmed by ^1^H NMR and FTIR spectroscopy. The ^1^H NMR spectrum of D6AB (Figure S1) clearly displayed characteristic signals corresponding to aromatic (Ar–H), methyl (Me–H), and acrylate (ACR–H) protons. FTIR analysis of the IPN-X elastomer (Figure 2a) further verified the completion of the cross-linking reactions: the disappearance of the characteristic peak of –C=C- at 1410 cm^–1^ indicates complete cross-linking of the acrylic ester groups; concomitantly, the absence of the −NCO peak at 2245 cm^–1^ signifies the formation of a polyurethane network. The crystalline structures of pure LCE and IPN-X elastomers were investigated using X-ray diffraction (XRD). As shown in Figure 2b, all samples exhibited broad diffraction peaks near 2θ = 20°, indicative of an amorphous structure. The incorporation of the PU network did not alter the amorphous nature of the LCE component.

**Figure 2:**
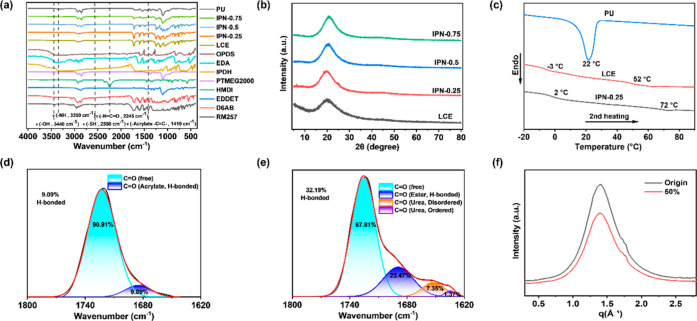
(a) FTIR spectrum. (b) XRD spectrum of LCE and IPN-X. (c) DSC curves of LCE, PU, and IPN samples. (d) C=O stretching vibration FTIR spectra of pure LCE. (e) C=O stretching vibration FTIR spectra of the IPN sample. (f) 1D-WAXS pattern of the IPN sample.

Subsequently, the mesophase properties of the samples were characterized by using differential scanning calorimetry (DSC) and wide-angle X-ray scattering (WAXS). As illustrated in Figure 2c, the pure LCE exhibits a glass transition temperature (*T*_g_) of −3 °C and a LC-to-isotropic phase transition temperature (*T*_iso_) of 52 °C.^17^ In the case of IPN-0.25, the *T*_g_ and *T*_iso_ temperatures increased to 2 and 72 °C, respectively. This upward shift can be attributed to the incorporation of PU chains into the LCE network, which causes its molecular chains to intercalate into the LCE network, affecting the ordered arrangement and motion of the LC units. Consequently, these units require higher temperatures to disrupt their ordered arrangement and the transition into an isotropic state. Additionally, the incorporation of PU also leads to changes in the hydrogen bond content.^26–30^ To further analyze changes in hydrogen bond content following IPN formation, Gaussian–Lorentzian fitting analysis was performed on characteristic peaks in the carbonyl region of the FTIR spectrum. As shown in Figure 2d,e, the hydrogen bond content of pure LCE is 9.09%, while after the formation of the IPN, the hydrogen bond content increases to 32.19%. This indicates an enhancement of hydrogen bonding interactions. Subsequently, wide-angle X-ray scattering (WAXS) experiments were conducted on the IPN samples, as shown in Figures 2f and S4. In the two-dimensional (2D) WAXS pattern, the initial IPN sample presented a completely circular ring, indicating that the polymers were randomly distributed without any internal orientation at this stage. Upon 50% stretching, the polymer chains began to align along the stretching direction under an external force, resulting in an arc-shaped contraction in the 2D-WAXS pattern. In the one-dimensional (1D) WAXS pattern, in comparison with the initial state, the IPN sample displays an internal orientation along the parallel tensile direction subsequent to stretching. This results in the attenuation of the diffraction signal perpendicular to the tensile direction and a decrease in the intensity of the main peak. The 1D-WAXS and 2D-WAXS patterns indicate that clear orientation alignment occurred within the IPN elastomers before and after stretching, enabling more efficient load transfer along the molecular chains, thereby contributing to the enhanced mechanical performance.

### 2.2 Mechanical Properties of IPN-X Elastomers

The enhancement of the mechanical properties of the LCE by the IPN is demonstrated by measuring the strain–stress curves of pure LCE and IPN-X elastomers. As demonstrated in Figure 3a, both strain and stress of the IPN-X elastomer gradually increase with rising PU content. This phenomenon can be attributed to the interpenetration of the flexible PU chains within the LCE following IPN formation. Consequently, when subjected to external forces, the material dissipates energy through mechanisms such as chain slip,^25,50,51^ thereby enhancing its fracture strain. Moreover, an increase in the PU content results in an enhancement of the structural density of the IPN, leading to an intensification of hydrogen bonding interactions and physical entanglement between molecular chains. This substantially enhances the material’s fracture strain and stress.^31–37^ It was observed that among the series of IPN-X elastomers, IPN-0.75 exhibited the most advanced properties, with a fracture strain of 1699% and the stress reaching 44 MPa while also demonstrating an excellent toughness of 307.43 kJ·m^–3^ (Figure S7). Consequently, IPN-0.75, exhibiting the highest stress and toughness, was selected as the primary subject of the investigation. Stress–relaxation curves were used to further investigate their mechanical properties. As shown in Figure 3b, the IPN-0.75 sample was tested at various strain levels. The results indicate that within 1200 s, the normalized stress of IPN-0.75 consistently exceeded 1/e.^40–49^ This demonstrates that no stress relaxation occurred during this period, indicating an excellent elastic retention capability. To clearly evaluate the toughness of IPN-0.75, a 1 mm notch was introduced, and tensile testing was conducted at a strain rate of 5 mm/min. The results demonstrated that the material retained a fracture strain of 962% and a stress of 15.9 MPa (Figure 3c). The fracture energy *G*_c_ of the material was calculated using the following formula

$$G_{c}=\frac{6WC}{\sqrt{\lambda_{c}}}$$

where *W* is the fracture toughness of the material, *C* represents the notch size, and λ_c_ indicates the fracture strain of the material after the notch. The fracture energy of IPN-0.75 was calculated to be 162.53 kJ·m^–2^.

**Figure 3:**
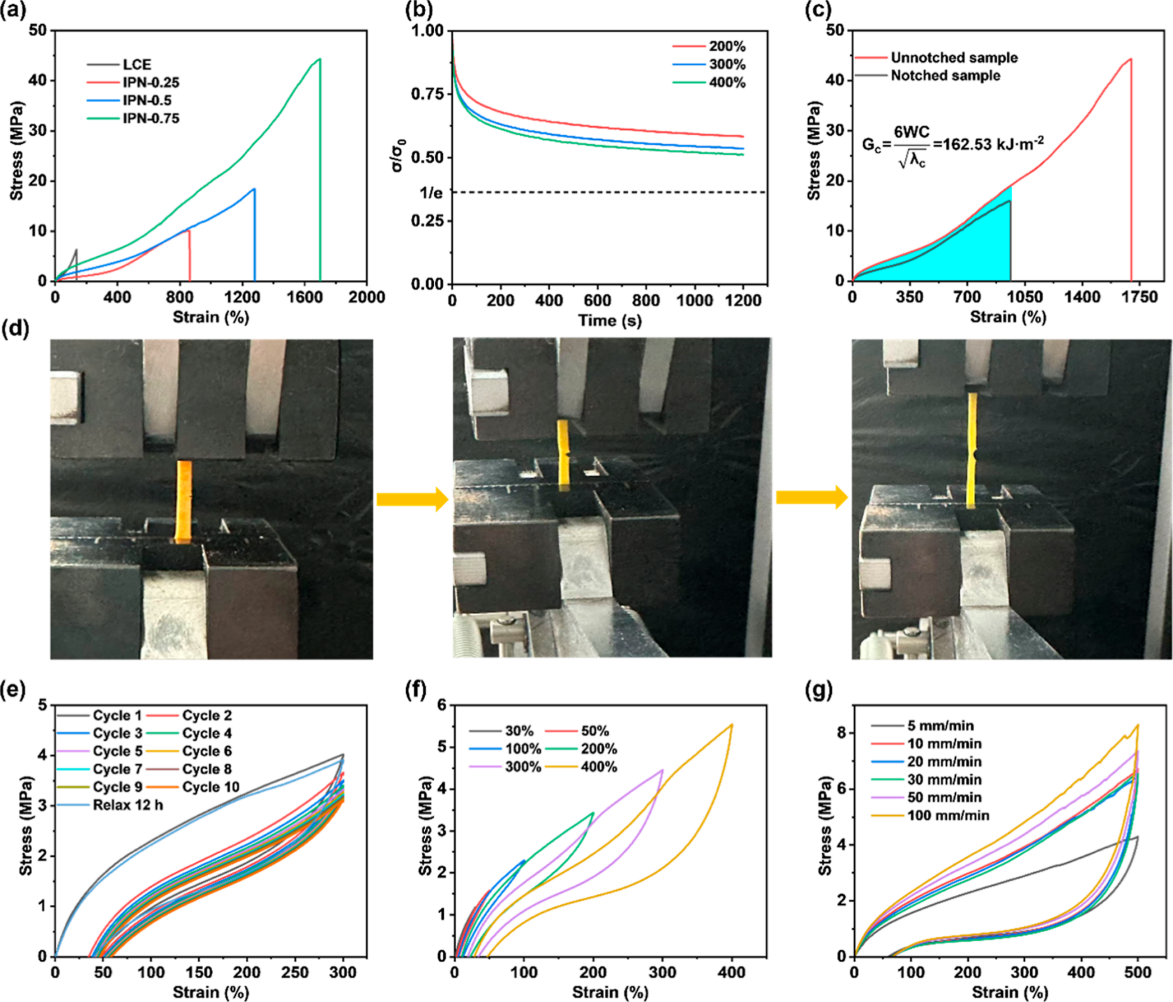
(a) Strain–stress curves of LCE and IPN-X elastomers. (b) Normalized stress–relaxation curves of IPN-0.75 at different strains (200%, 300%, and 400%). (c) Fracture energy of IPN-0.75. (d) Fracture energy testing procedure. (e) Cyclic tensile curve of IPN-0.75 over ten cycles at 300% strain. (f) Cyclic tensile curves of IPN-0.75 with different strains at 50 mm/min tensile speed. (g) Cyclic tensile curves of IPN-0.75 with different tensile speeds at 500% strain.

Subsequently, the cyclic tensile behavior of IPN-0.75 was evaluated to assess its fatigue resistance and mechanical stability. As demonstrated in Figure 3e, the sample was subjected to 10 consecutive loading–unloading cycles at a fixed strain of 300%. The cyclic tensile curve of the material demonstrated stability over 4–10 cycles, indicating internal mechanical stabilization. After 12 h of relaxation, the tensile curve nearly returned to its original state, demonstrating the structural recovery capability of IPN-0.75 through molecular chain thermal motion and chain rearrangement. At a constant tensile rate of 50 mm/min, cyclic tensile curves were tested at different strains, as shown in Figure 3f. As the maximum strain increased, the peak stress of IPN-0.75 also increased, indicating that the material possesses a certain ability to resist deformation. Furthermore, to investigate the mechanical properties of IPN-0.75 under dynamic loading, cyclic tensile curves were obtained at different tensile rates. As demonstrated in Figure 3g, at a constant strain of 500%, the peak stress of the material increases clearly with an increasing tensile rate. This phenomenon occurs because, at low tensile rates, molecular chains have sufficient time to relax stress through mechanisms such as chain slip. As the tensile rate increases, molecular chains are unable to relax stress in time, resulting in stress concentration within the molecules and a subsequent rise in peak stress. A series of strain–stress diagrams for the material demonstrates its outstanding mechanical properties, establishing the foundation for its stability under photoresponsive driving.

### 2.3 Photo-responsive Behavior of IPN-X Elastomers

The photoresponsive behavior of the IPN-X elastomers originates from the incorporation of azobenzene units into the polymer architecture. As illustrated in Figure 4a, during thermal stretching at 70 °C, the molecular chains within the elastomers transited from a disordered structure to a uniformly oriented structure. Upon exposure to 365 nm ultraviolet (UV) light, the azobenzene exhibited trans-to-cis isomerization, resulting in the generation of stress differences within the elastomer and leading to its bending. The process of azobenzene isomerization was investigated using an ultraviolet–visible spectrum (Figure 4b,c). Taking IPN-0.75 as an example, the spectrum shows two absorption peaks at 360 and 450 nm, corresponding, respectively, to the π → π* transition of the –N=N- group in *trans*-azobenzene and the *n* → π* transition of the –N=N- group in *cis*-azobenzene.^38,39^ Under UV irradiation, the absorption peak at 360 nm decreased, while the peak at 450 nm increased, corresponding to the isomerization of azobenzene from trans to the cis form. After exposure to visible light, a rise in the absorption peak at 360 nm was observed, while the peak at 450 nm decreased, indicating *cis*-to-trans back-isomerization of azobenzene.

**Figure 4:**
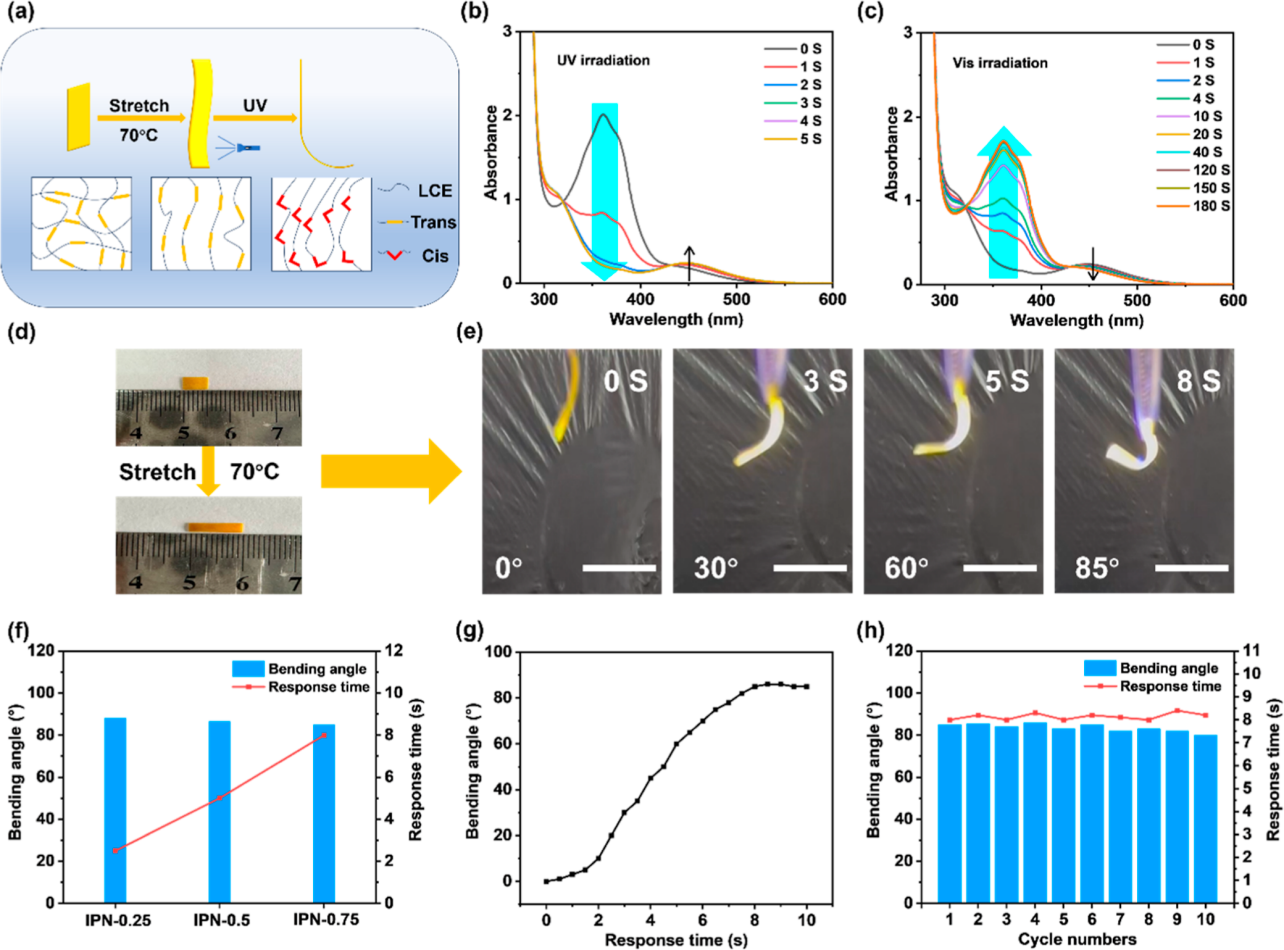
(a) Isomerization process of azobenzene. (b) UV absorption spectrum of IPN-0.75. (c) Vis absorption spectrum of IPN-0.75. (d) Thermal stretching of IPN-0.75. (e) Photoresponsive bending process of IPN-0.75. (f) Bending angle–response time diagrams for different proportions of IPN-X. (g) Response time–bending angle curve of IPN-0.75. (h) Cyclic UV irradiation diagram for IPN-0.75.

After thermal treatment, the IPN-0.75 film (5 × 1 × 0.2 mm) exhibited reversible photoinduced bending upon 365 nm UV (50 mW/cm^2^) irradiation, attaining a maximum bending angle of 85° after 8 s (Figure 4d,e). Under consistent illumination conditions, the bending performance of IPN-X films with varying PU contents was evaluated. As summarized in Figure 4f, while the maximum bending angle remained largely unaffected by the PU content, the response time increased progressively. This delay is attributed to the increased cross-linking density and restricted chain mobility induced by a higher PU content, which slows the propagation of material deformation triggered by azobenzene isomerization and thus prolongs the film’s photo-response time. In consideration of the fact that the maximum bending angles of IPN-X films with different ratios are similar, IPN-0.75 was chosen for subsequent investigations due to its superior mechanical properties.

The bending angle of IPN-0.75 as a function of the response time is demonstrated in Figure 4g. Within the initial 2 s of irradiation, azobenzene begins to isomerize; however, the microstructural changes are inadequate to induce macroscopic deformation. Between 2 and 8 s, extensive azobenzene isomerization occurs, causing rapid material bending. After irradiation for 8 s, the azobenzene isomerization reaction reaches equilibrium, and the material’s bending enters a saturated stage, at which point no further obvious changes occur. To investigate the photostability of IPN-0.75, the material underwent 10 cycles of cyclic UV exposure under the same UV intensity. As demonstrated in Figure 4h, over a period of 10 cycles, the bending angle and response time of IPN-0.75 remained essentially constant, indicating excellent photostability. This property is of particular significance for its practical application in photoresponsive devices.

### 2.4 Thermal Properties, Recyclability, and Triple Shape Memory Effect of IPN-X Elastomers

For IPN-X elastomers, thermal properties are one of the key indicators for evaluating material performance and guiding their application scenarios. Thermogravimetric analysis (Figure 5a) reveals that all three IPN-X films exhibit a thermal decomposition temperature (*T*_d_) of 300 °C and a complete decomposition temperature exceeding 600 °C, demonstrating their excellent thermal stability. The DSC curves of IPN-X films (Figure 5b,c) reveal that the phase transitions of the three materials are similar, with a glass transition temperature (*T*_g_) around −6 °C and an LC-to-isotropic phase transition temperature (*T*_iso_) around 70 °C. Taking IPN-0.75 as an example, its polarized optical microscopy (POM) image (Figure S2) shows that the material undergoes a distinct structural transition near 72 °C, consistent with the results revealed by the DSC curve.

**Figure 5:**
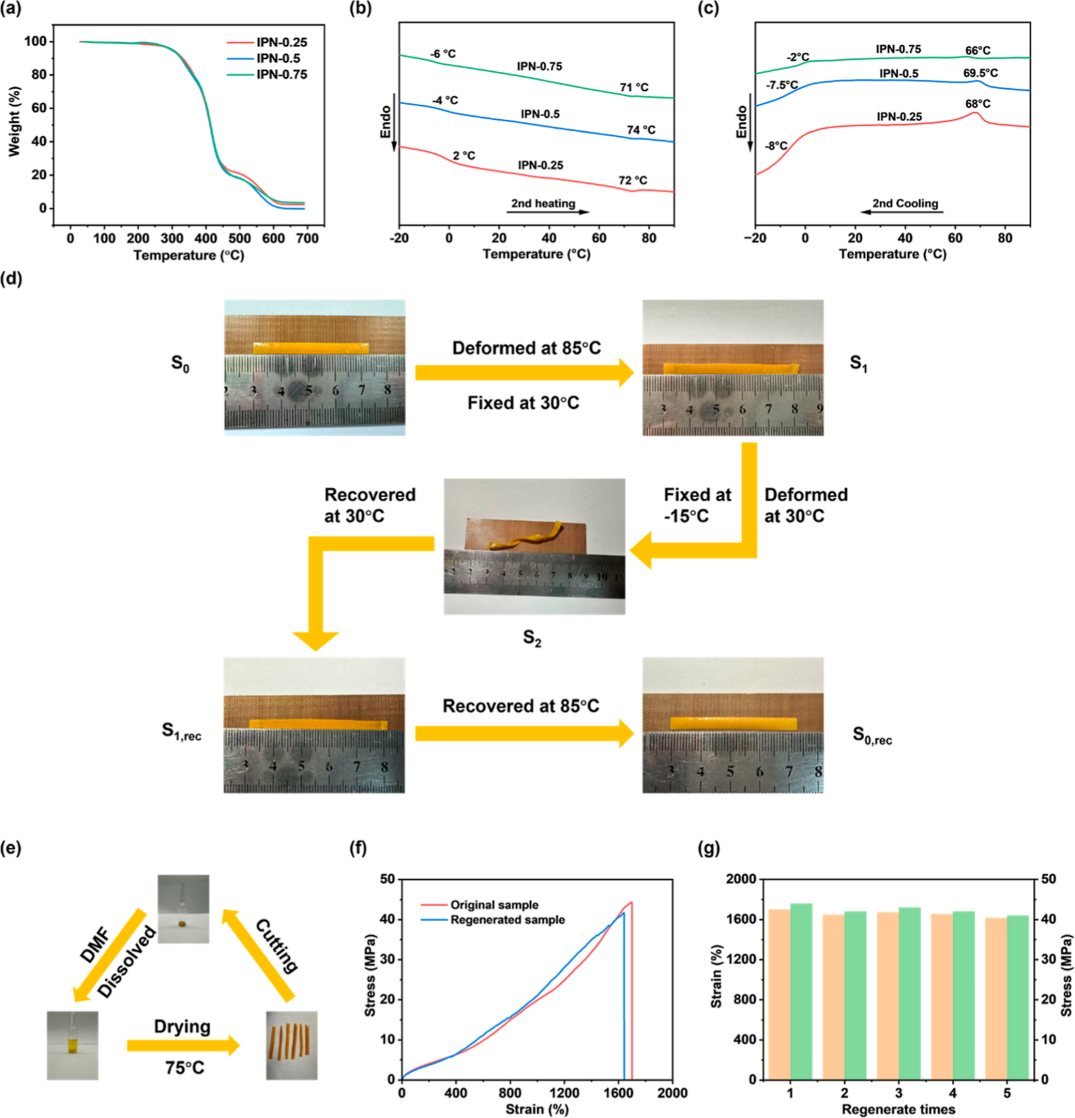
(a) TGA curves of IPN-X elastomers. (b) Heating DSC curves of IPN-X elastomers. (c) Cooling DSC curves of IPN-X elastomers. (d) Triple shape memory effect (TSME) of IPN-0.75. (e) Regeneration process of IPN-0.75. (f) Strain–stress curves of original and regenerated IPN-0.75. (g) Cyclic regeneration performance diagram for IPN-0.75.

Based on the above analysis, a triple shape memory effect (TSME) was designed for the IPN-0.75 film. As demonstrated in Figure 5d, the film was subjected to a tensile strain at 85 °C (above *T*_iso_) and subsequently cooled to 30 °C to establish the temporary shape S_1_. Subsequently, the stretched film was curled into a spiral shape and cooled to −15 °C (below *T*_g_) to fix the shape of S_2_. After fixation, the film was heated to 30 °C, where helical unwinding was observed as the film reverted to its temporary shape S_1_. Subsequently, heating to 85 °C caused the film to thermally shrink, restoring it to its original shape S_0_. These TSME properties illustrate the precise controllability and durability in their “deformation-recovery” behavior.

To further investigate the durability of the film, IPN-0.75 was selected as the subject. As shown in Figure S9, IPN-0.75 swells but does not dissolve in DCM. The highly polar solvent, DMF, has the capacity to disrupt hydrogen bonds between molecular chains and is selected for use as a recycling solvent. The film was shredded, dissolved in DMF, dried at 75 °C to remove the solvent, and reprocessed to obtain a recycled film (Figure 5e). Tensile tests conducted on the regenerated film, as shown in Figure 5f, revealed no obvious decrease in fracture strain or stress compared to the original film. Following five cycles of dissolution–regeneration, no obvious reduction was observed in either the fracture strain or the fracture stress of the film, as shown in Figure 5g. This finding suggests that the material exhibits excellent recyclability, enabling multiple regenerations without substantial performance degradation. The properties of this material provide strong support for its application in sustainable materials and recycling scenarios.

## 3 Conclusion

In summary, we have successfully developed a light-responsive liquid crystal elastomer (IPN-0.75) with outstanding mechanical properties, exhibiting a maximum fracture strain of 1699% and a maximum stress of 44 MPa. Furthermore, IPN-0.75 demonstrates excellent recyclability, exhibiting a tensile strength of 42 MPa after five consecutive “dissolution–regeneration” cycles. Moreover, IPN-0.75 serves as an excellent photoresponsive actuator, exhibiting an 85° bend within 8 s under 365 nm UV irradiation. Its photoresponsive actuation properties remain largely unchanged over 10 cycles, demonstrating outstanding stability. This photoresponsive LCE material, which exhibits exceptional tensile strength and stability, overcomes the mechanical weakness present in traditional LCE materials. It is evident that this technology has great potential for application in a range of fields including soft robotics and bionic actuation.

## 4 Experimental Section

### 4.1 Preparation of the IPN-0.75 Elastomer

First, PTMEG ∼2000 was vacuum-dried for 2 h. Then, HMDI (235.95 mg, 0.9 mmol), PTMEG ∼2000 (750 mg, 0.375 mmol), and DBTBL (5 mg) were dissolved in 5 mL of DMAc. Stir the solution at 75 °C under a nitrogen atmosphere for 2 h to complete the prepolymerization; Concurrently, RM257 (176.6 mg, 0.3 mmol), EDDET (27.35 mg, 0.15 mmol), D6AB (23.51 mg, 0.045 mmol), and BAPO (10 mg) were dissolved in 3 mL DMAc. TEA (5 mg) was added, and the mixture was stirred at room temperature for 6 h to perform the Michael addition reaction. After completion of the prepolymerization, IPDH (36.41 mg, 0.1875 mmol) and EDA (39.8 mg, 0.1875 mmol) were dissolved in 5 mL of DMAc and added to the PU oligomer solution. The solution was stirred at 45 °C for 2 h. Subsequently, OPDS (37.55 mg, 0.15 mmol) was added and stirred at 75 °C for 2 h. After the reaction was complete, the PU solution was added to the LC oligomer and stirred for 10 min to initiate dual-network cross-linking. After stirring, the solution was exposed to 365 nm UV light (50 mW/cm^2^) for 1 min to ensure that acrylic ester groups were completely cross-linked within the PU network. Finally, the solution was placed in a PTFE mold and vacuum-dried at 70 °C to produce the IPN-0.75 film.

### 4.2 Preparation of the IPN-0.25 Elastomer

First, PTMEG ∼2000 was vacuum-dried for 2 h. Then, HMDI (78.71 mg, 0.3 mmol), PTMEG ∼2000 (250 mg, 0.125 mmol), and DBTBL (5 mg) were dissolved in 5 mL of DMAc. The solution was stirred at 75 °C under a nitrogen atmosphere for 2 h to complete the prepolymerization; concurrently, RM257 (529.74 mg, 0.9 mmol), EDDET (82.04 mg, 0.45 mmol), D6AB (70.54 mg, 0.135 mmol), and BAPO (10 mg) were dissolved in 3 mL of DMAc. TEA (5 mg) was added, and the mixture was stirred at room temperature for 6 h to carry out the Michael addition reaction. After completion of the prepolymerization, IPDH (12.14 mg, 0.0625 mmol) and EDA (13.27 mg, 0.0625 mmol) were dissolved in 5 mL of DMAc and added to the PU oligomer solution. The solution was stirred at 45 °C for 2 h. Subsequently, OPDS (12.52 mg, 0.05 mmol) was added, and the mixture was stirred at 75 °C for 2 h. After the reaction was complete, the PU solution was added to the LC oligomer and stirred for 10 min to initiate dual-network cross-linking. After stirring, expose the solution to 365 nm UV light (50 mW/cm^2^) for 1 min to ensure that acrylic ester groups are completely cross-linked within the PU network. Finally, the solution was placed in a PTFE mold and vacuum-dried at 70 °C to produce the IPN-0.25 film.

### 4.3 Preparation of the IPN-0.5 Elastomer

First, PTMEG ∼2000 was vacuum-dried for 2 h. Then, HMDI (157.42 mg, 0.6 mmol), PTMEG ∼2000 (500 mg, 0.25 mmol), and DBTBL (5 mg) were dissolved in 5 mL of DMAc. The solution was stirred at 75 °C under a nitrogen atmosphere for 2 h to complete the prepolymerization; concurrently, RM257 (353.16 mg, 0.6 mmol), EDDET (54.69 mg, 0.3 mmol), D6AB (47.03 mg, 0.09 mmol), and BAPO (10 mg) were dissolved in 3 mL of DMAc. TEA (5 mg) was added, and the mixture was stirred at room temperature for 6 h to carry out the Michael addition reaction. After completion of the prepolymerization, IPDH (24.27 mg, 0.125 mmol) and EDA (26.54 mg, 0.125 mmol) were dissolved in 5 mL of DMAc and added to the PU oligomer solution. The solution was stirred at 45 °C for 2 h. Subsequently, OPDS (25.03 mg, 0.1 mmol) was added, and the mixture was stirred at 75 °C for 2 h. After the reaction was complete, the PU solution was added to the LC oligomer and stirred for 10 min to initiate dual-network cross-linking. After stirring, the solution was exposed to 365 nm UV light (50 mW/cm^2^) for 1 min to ensure that acrylic ester groups were completely cross-linked within the PU network. Finally, the solution was placed in a PTFE mold and vacuum-dried at 70 °C to produce the IPN-0.5 film.

### 4.4 Recycling and Actuation Methods

The IPN-0.75 elastomer was ground and dissolved in 5 mL of DMF. The mixture was placed in a PTFE mold and dried at 75 °C to remove the solvent; then, the recycled film was obtained.

For the actuation experiment, IPN-0.75 was first thermally stretched at 70 °C for 30 s and then irradiated with 365 nm UV (50 mW/cm^2^) light to observe its driving process.

### 4.5 Characterization

^1^H Nuclear Magnetic Resonance (NMR) spectroscopy was measured on an Avance III HD 400 MHz spectrometer (Bruker, Germany). Fourier transform infrared (FTIR) spectroscopy was performed on an INVENIO S spectrometer (Bruker, Germany). The thermogravimetric analysis (TGA) spectrum was measured on a TGA Q50 (TA INSTRUMENTS, America). Differential Scanning Calorimetry (DSC) was measured on a DISCOVERY DSC 250 (TA INSTRUMENTS, America). Mechanical tests were performed on a UTM 2502 instrument (Sansi, China). Polarizing microscopy (POM) images were measured on a DMRX instrument (Leica, Germany). Wide-Angle X-ray Scattering (WAXS) was measured on a Xeuss 2.0 instrument (Xenocs, France).

All characterizations and performance tests were conducted on five independent parallel samples.

### 4.6 Analysis Methods

#### 4.6.1 Differential Scanning Calorimetry

The test was conducted under a nitrogen atmosphere. Measurements were conducted within the temperature range of −40 to 200 °C at a heating rate of 5 °C/min, followed by cooling back to −40 °C and reheating to 200 °C.

#### 4.6.2 Thermogravimetric Analysis

The test was conducted in a nitrogen atmosphere. Measurements were performed at a heating rate of 5 °C/min within the temperature range of 25–700 °C.

#### 4.6.3 Tensile Test

Rectangular samples (5 × 1 × 0.2 mm) were tested for tensile strength at a rate of 50 mm/min until fracture occurred.

#### 4.6.4 Normalized Stress–Relaxation Test

Rectangular samples (5 × 1 × 0.2 mm) were stretched at a rate of 50 mm/min to a set strain and held for 1200 s.

#### 4.6.5 X-ray Diffraction Analysis

The test was conducted at a scanning rate of 5°/min, with the scanning angle ranging from 2θ = 5° to 85°.

## Supplementary Material





## Notes

Synthesis procedures of all samples, ^1^H NMR spectrum of D6AB, POM images, SEM images, 2D-WAXS images, FTIR spectra, DSC curves of all samples, swelling experiment, weight-load, and puncture–resistance tests (PDF)
Ultraviolet photoresponsive actuation of the IPN-0.75 elastomer (MP4)
