# Supplementary material for: Mechanically Robust Photo-responsive Liquid Crystal Elastomers
Source: ACS Omega. 2026 Apr 9;11(15):23595–603. doi: 10.1021/acsomega.6c02486 (PMC13103854; doi:10.1021/acsomega.6c02486)
Supplement: Supplementary file 1 [file ao6c02486_si_001.pdf]

Supporting information

Mechanically Robust Photo-Responsive Liquid Crystal Elastomer

*Xitong Dong, Kuohuai Qin, Hao Ouyang, Zhi-Hui Ren, and Zheng-Hui Guan**

Key Laboratory of Synthetic and Natural Functional Molecule of the Ministry of Education, College of Chemistry & Materials Science Northwest University,
Xi'an 710127, P. R. China
E-mail: guanzhh@nwu.edu.cn

Tabel of Contents

1. Materials. 3

2. Synthesis and characterizations of D6AB.......... 3

3. Synthesis and preparation of pure LCE. 3

4. Synthesis and preparation of pure PU.......... 4

5. Supporting Figures and Tables. 4

6. References.......... 10

1. Materials.

4,4'-bis(6-hydroxyhexyloxy)azobenzene was purchased from Zhengzhou Huiju Chemical Co., Ltd. LC monomer RM257, triethyl-amine (TEA), 3,6-dioxa-1,8-octanedithiol (EDDET) and photoinitiator BAPO were purchased from Shanghai Bepharm Science & Technology Co., Ltd. Isocyanate (HMDI), chain extender isophthalic dihydrazide (IPDH), 4,4'-ethylenedianiline (EDA), and 4,4'-dihydroxydiphenyl disulfide (OPDS) were purchased from Anhui Zesheng Technology Co., Ltd.

2. Synthesis and characterizations of D6AB.

Synthesis of D6AB: D6AB was synthesized via an acylation reaction between 4,4'-bis(6-hydroxyhexyloxy)azobenzene and acryloyl chloride. First, dissolve 4,4'-bis(6-hydroxyhexyloxy)azobenzene (0.415 g, 1 mmol) in 20 mL of THF, add TEA (0.202 g, 2 mmol) into the solution and stirred at 0°C for 30 min. After stirring is complete, dissolve acryloyl chloride (0.181 g, 2 mmol) in 8 mL of THF and slowly add it to the solution at 0°C. After the addition is complete, stir the solution at room temperature for 24 hours. After the reaction is complete, the mixture is concentrated to obtain the crude product. The crude product was purified through a silica gel column with petroleum ether/ethyl acetate (1/1) as the eluent. The product is a yellow powder. Yield: 41%.

^{1}H NMR (400 MHz, $CDCl_3$) δ 7.86 (d, J = 8.8 Hz, 4H), 6.98 (d, J = 8.9 Hz, 4H), 6.40 (dd, J = 17.4, 1.5 Hz, 2H), 6.12 (dd, J = 17.4, 10.4 Hz, 2H), 5.82 (dd, J = 10.4, 1.5 Hz, 2H), 4.18 (t, J = 6.6 Hz, 4H), 4.03 (t, J = 6.4 Hz, 4H), 1.72 (s, 4H), 1.47 (s, 12H).

3. Synthesis and preparation of pure LCE.

Dissolve RM257 (353.2 mg, 0.6 mmol), EDDET (54.69 mg, 0.3 mmol), D6AB (47.03 mg, 0.09 mmol) and BAPO in 3 mL DMAc. Add 5 mg TEA and stir the solution at room temperature for 6 hours to carry out the Michael addition reaction. After completion, expose the solution to 365 nm UV light for 1 minute to react the remained acrylate groups. Finally, place the polymer solution in a PTFE mold and vacuum-dry it at 70°C to remove the solvent to obtain the LCE film.

4. Synthesis and preparation of pure PU.

Dissolve HMDI (157.42 mg, 0.6 mmol) and PTMEG~2000 (500 mg, 0.25 mmol) in 6 mL DMAc. Subsequently, add 5 mg DBTDL and stir the solution for 2 h at 75°C under a nitrogen atmosphere. Upon completion, IPDH (24.27 mg, 0.125 mmol), EDA (26.54 mg, 0.125 mmol) and OPDS (25.03 mg, 0.1 mmol) were sequentially added. The mixture was stirred at 75°C under a nitrogen atmosphere for 2 hours to complete the reaction. Finally, the polymer solution is placed in a PTFE mold and vacuum-dried at 70°C to remove the solvent to obtain the PU film.

5. Supporting Figures and Tables.

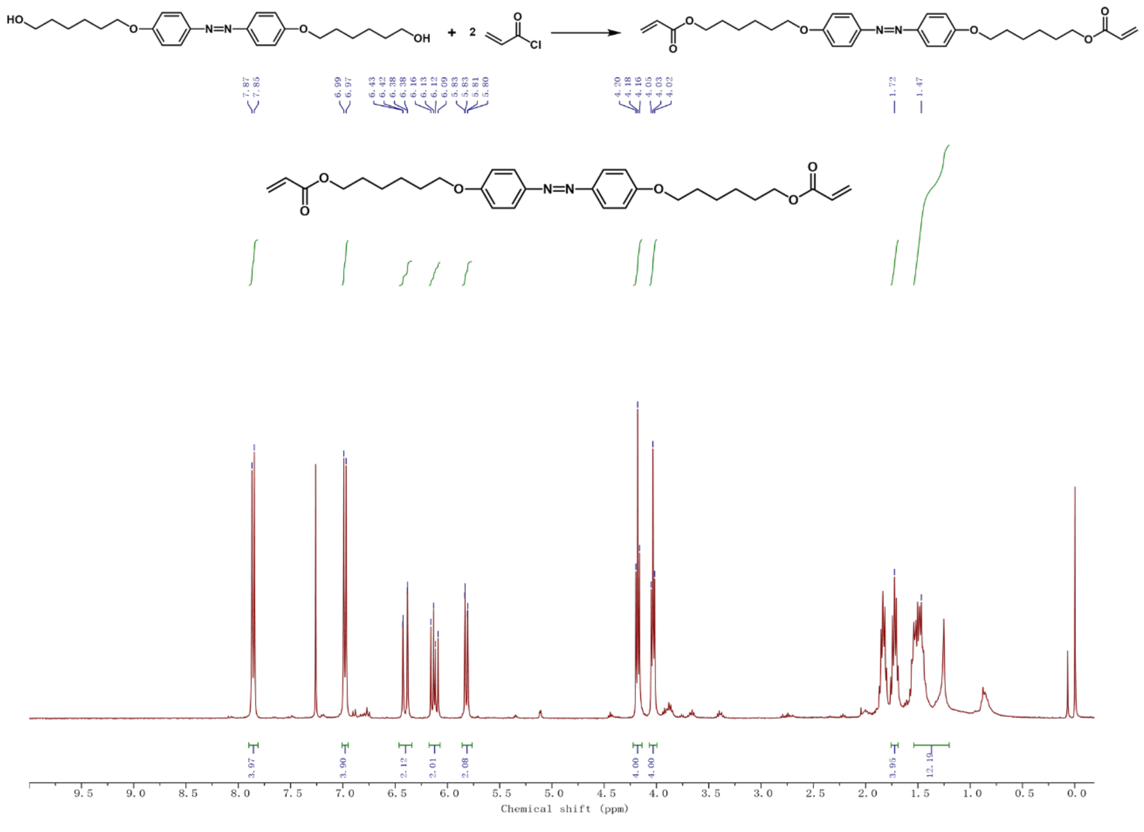

Figure S1. ^{1}H NMR spectrum of D6AB.

In order to investigate the liquid crystal phase transition behavior of the elastomer, Polarized Optical Microscopy (POM) images were captured at different temperatures. The elastomer demonstrated discernible textural alterations in the proximity of the

phase transition temperature (near 71°C), which was corroborated by Differential Scanning Calorimetry (DSC) curves.

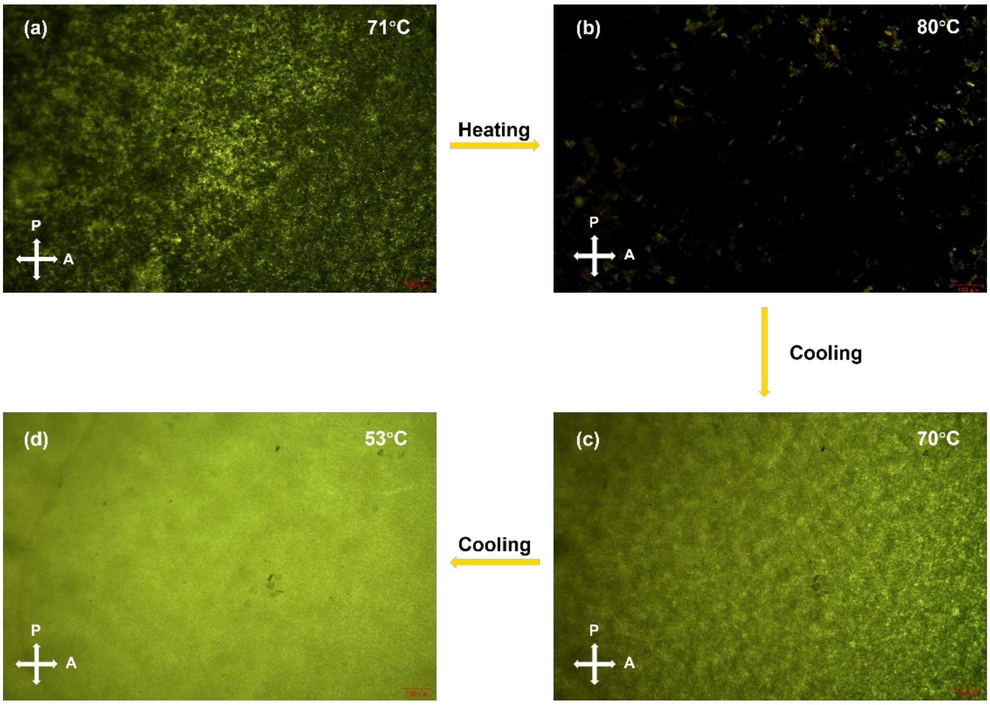

Figure S2. POM images of IPN-0.75 at different temperatures.

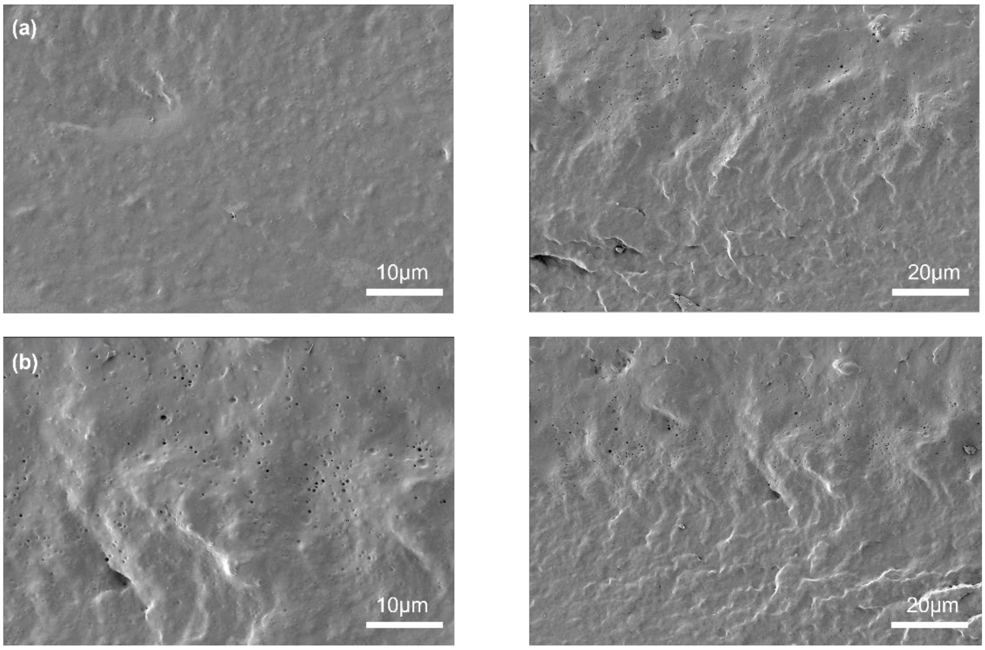

Figure S3. SEM images of IPN-0.75 (a) before recycling, (b) after recycling.

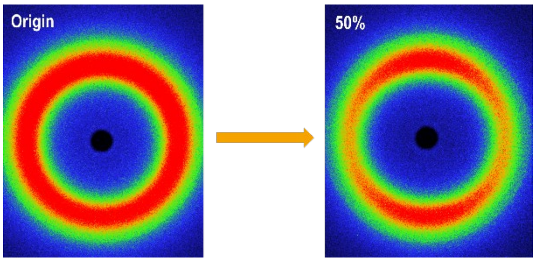

Figure S4. 2D-WAXS images of IPN-0.75.

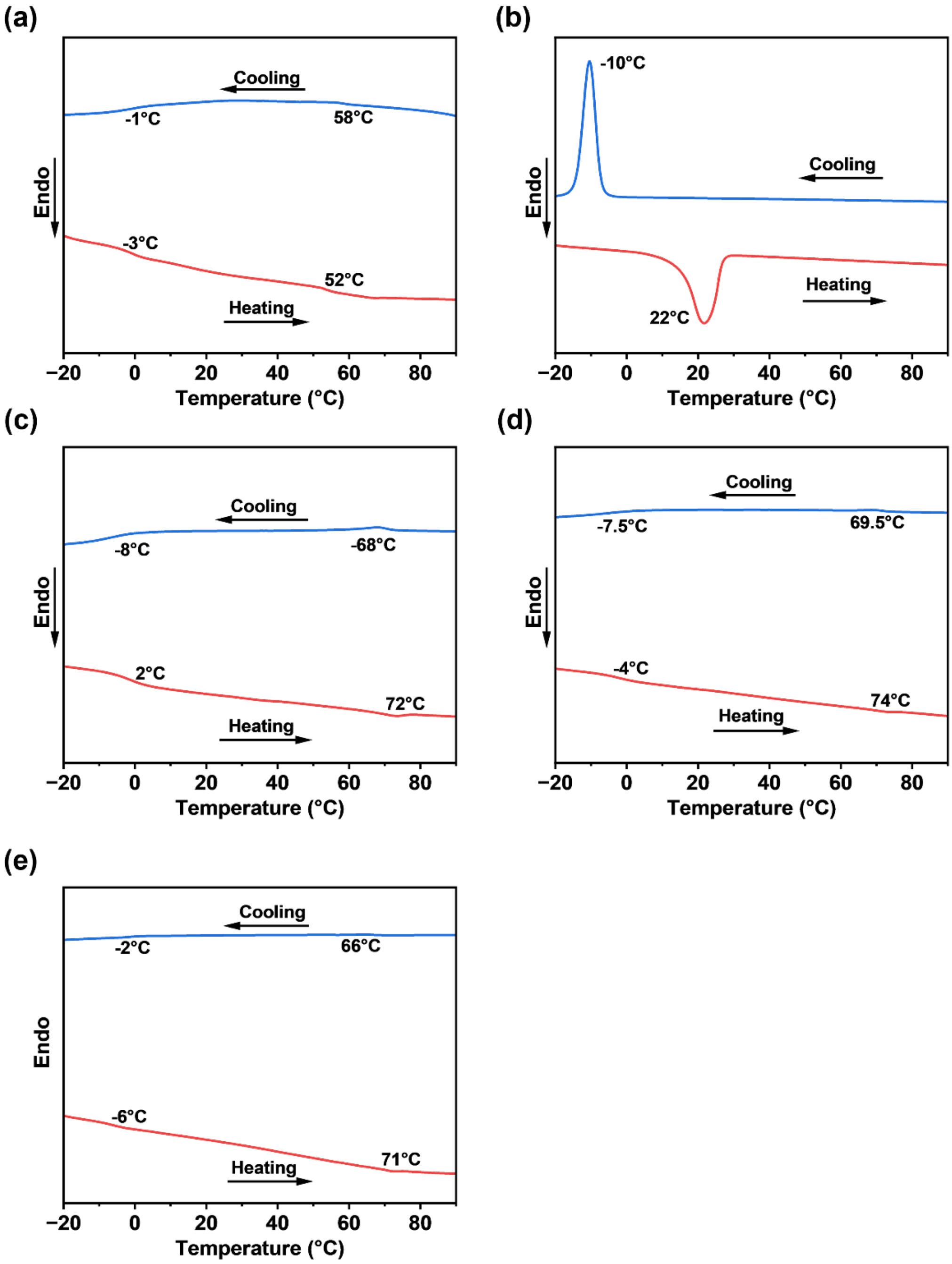

Figure S5. The DSC curves of (a) LCE, (b) PU, (c) IPN-0.25, (d) IPN-0.5, (e) IPN-

0.75.

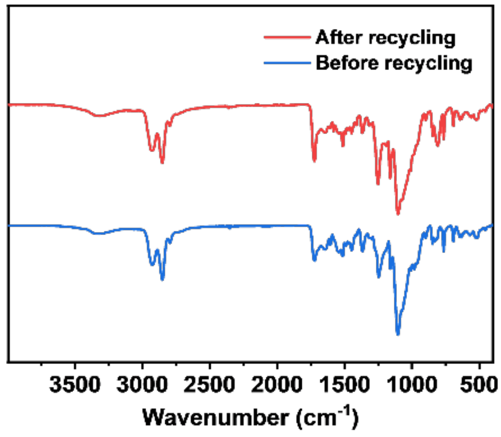

Figure S6. FTIR spectra of IPN-0.75 in different states.

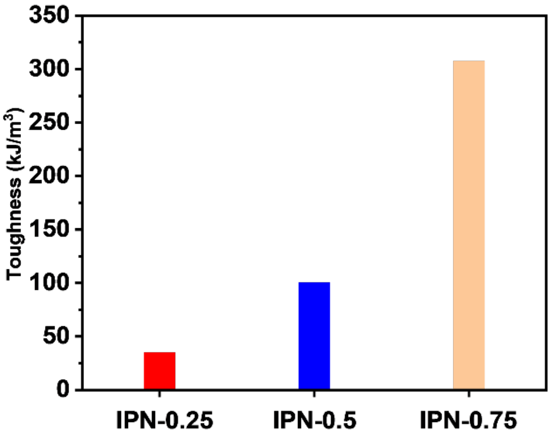

Figure S7. Toughness of IPN-X elastomers.

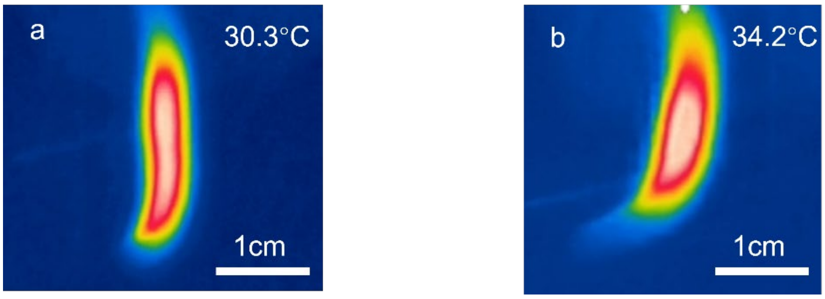

Figure S8. Infrared thermal image of IPN-0.75 in (a) under UV irradiation, (b) after UV irradiation.

Swelling Experiment

The dimensions and weight of original IPN-0.75 sample were 19.6 mm long × 13.1 mm wide × 0.32 mm thick and 34.4 mg. After swollen in dichloromethane (DCM) for 12 hours, the dimensions and weight changed to 38.3 mm long × 25.2 mm wide × 0.41 mm thick and 70.7 mg. Subsequently, IPN-0.75 was vacuum dried at 60°C for 12 hours, after which its weight decreased to 31.7 mg. Swelling degree (S_d) and erosion rate (E_r) of IPN-0.75 were calculated using the following formulas:

$$S_d=\frac{m_1\text{-}m_0}{m_0}\times 100\% \quad (1)$$

$$E_r=\frac{m_0\text{-}m_2}{m_0}\times 100\% \quad (2)$$

where m_0 is the original weight of IPN-0.75, m_1 is the swollen weight, and m_2 is the weight of the dried IPN-0.75. Calculations show that IPN-0.75 exhibits a swelling degree of 105.52% and erosion rate of 7.85%.

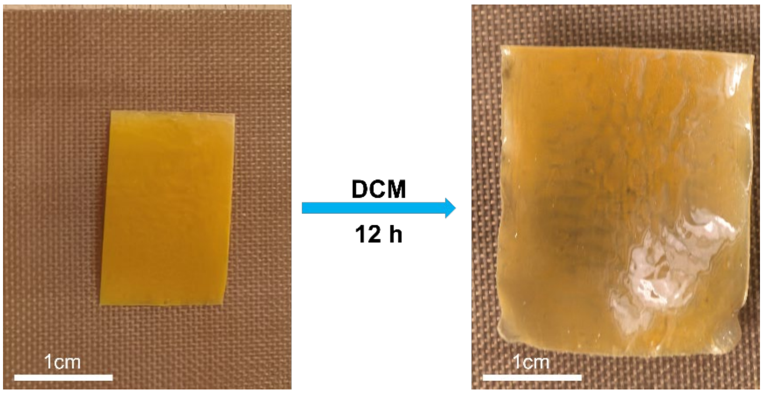

Figure S9. Swelling experiment of IPN-0.75 Elastomer.

Weight-load and Puncture-Resistance Tests

To further investigate the mechanical properties of IPN-0.75, we conducted weight load and puncture resistance tests. A rectangular sample weighing 140 mg was utilized in the experimental procedure, and it successfully lifted a 10 kg weight. The mass of the weight is found to be 7.14×10^4 times that of the sample.

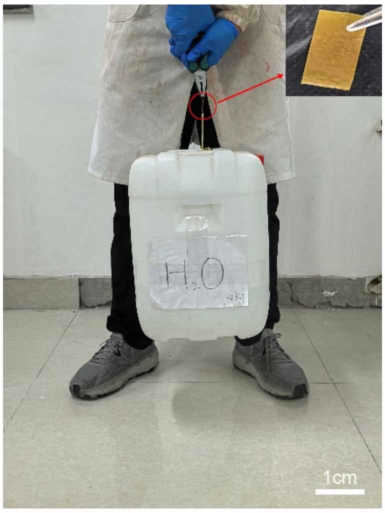

Figure S10. Weight-load image of IPN-0.75 Elastomer.

Additionally, we conducted a puncture resistance test on another rectangular sample. The results showed that the sharp object did not pierce through the sample but instead left a dot on its surface.

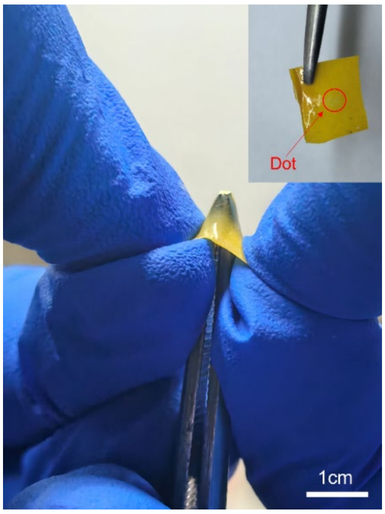

Figure S11. Puncture-Resistance Test of IPN-0.75 Elastomer.

Table S1. Chemical formulations (mg) for IPN-X Elastomers.

Component	Reagents	IPN-0.25	IPN-0.5	IPN-0.75
LCE	RM257	529.74	353.16	176.58
	EDDET	82.04	54.69	27.35
	D6AB	70.54	47.03	23.51
	BAPO	10	10	10
	TEA	5	5	5
PU	HMDI	78.71	157.42	235.95
	PTMEG~2000	250	500	750
	IPDH	12.14	24.27	36.41
	EDA	13.27	26.54	39.80
	OPDS	12.52	25.03	37.55
	DBTDL	5	5	5

6. References.

(1) Jang, M.; Kim, J. S.; Kim, J. H.; Bae, D. H.; Kim, M. J.; Son, D.; Kim, Y. T.; Um, S. H.; Kim, Y. H.; Kim, J. Surface-Controlled Molecular Self-Alignment in Polymer Actuators for Flexible Microrobot Applications. Polymers 2019, 11 (4), 736.

(2) Cui, Z.; Yue, X.; Wang, Y.; Zhang, Y.; Ren, Z.-H.; Guan, Z.-H. A light-responsive poly(urethane-urea) actuator with room temperature self-healing performance. Chem. Eng. J. 2024, 479, 147538.

(3) Ren, Z.; Zhang, Y.; Dong, C.; Li, N.; Cui, Z.; Guan, Z. Light- and heat-induced shape deformation polyurethane actuators that combine self-healing capabilities. Mater. Today Commun. 2024, 39, 109102.
